# Bis{4-bromo-2-[(2-hy­droxy­eth­yl)imino­meth­yl]phenolato}nickel(II) monohydrate

**DOI:** 10.1107/S1600536811031771

**Published:** 2011-08-11

**Authors:** Chen-Yi Wang, Jing-Fen Li, Ping Wang, Cai-Jun Yuan

**Affiliations:** a Department of Chemistry, Huzhou University, Huzhou 313000, People’s Republic of China; b College of Chemical Engineering, Nanjing Forestry University, Nangjing 210037, People’s Republic of China

## Abstract

The title mononuclear nickel complex, [Ni(C_9_H_9_BrNO_2_)_2_]·H_2_O, was obtained by the reaction of 5-bromo­salicyl­aldehyde, 2-amino­ethanol and nickel nitrate in methanol. The Ni^II^ atom is six-coordinated by two phenolate O, two imine N and two hy­droxy O atoms from two crystallographically different Schiff base ligands, forming an octa­hedral geometry. In the crystal, mol­ecules are linked by inter­molecular O—H⋯O and O—H⋯Br hydrogen bonds, forming a three-dimensional network.

## Related literature

For urease inhibitors, see: Wang (2009 ▶); Wang & Ye (2011 ▶). For related nickel(II) complexes, see: Arıcı *et al.* (2005 ▶); Liu *et al.* (2006 ▶); Li & Wang (2007 ▶); Ali *et al.* (2006 ▶).
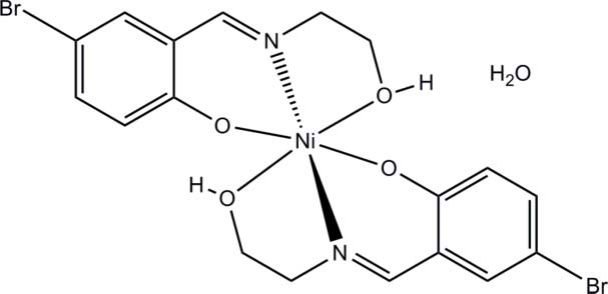


## Experimental

### 

#### Crystal data

[Ni(C_9_H_9_BrNO_2_)_2_]·H_2_O
*M* *_r_* = 562.89
Orthorhombic, 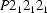

*a* = 9.835 (3) Å
*b* = 12.851 (2) Å
*c* = 16.226 (3) Å
*V* = 2050.8 (8) Å^3^
*Z* = 4
Mo *K*α radiation
μ = 4.87 mm^−1^
*T* = 298 K
0.21 × 0.20 × 0.20 mm

#### Data collection

Bruker SMART CCD area-detector diffractometer
Absorption correction: multi-scan (*SADABS*; Sheldrick, 1996 ▶) *T*_min_ = 0.428, *T*_max_ = 0.442
13318 measured reflections
4474 independent reflections
2310 reflections with *I* > 2σ(*I*)
*R*_int_ = 0.099

#### Refinement

*R*[*F* ^2^ > 2σ(*F* ^2^)] = 0.048
*wR*(*F* ^2^) = 0.128
*S* = 1.02
4474 reflections
259 parameters
3 restraints
H atoms treated by a mixture of independent and constrained refinement
Δρ_max_ = 0.60 e Å^−3^
Δρ_min_ = −0.95 e Å^−3^
Absolute structure: Flack (1983 ▶), 1930 Friedel pairs
Flack parameter: 0.013 (19)

### 

Data collection: *SMART* (Bruker, 1998 ▶); cell refinement: *SAINT* (Bruker, 1998 ▶); data reduction: *SAINT*; program(s) used to solve structure: *SHELXS97* (Sheldrick, 2008 ▶); program(s) used to refine structure: *SHELXL97* (Sheldrick, 2008 ▶); molecular graphics: *SHELXTL* (Sheldrick, 2008 ▶); software used to prepare material for publication: *SHELXTL*.

## Supplementary Material

Crystal structure: contains datablock(s) global, I. DOI: 10.1107/S1600536811031771/ci5196sup1.cif

Structure factors: contains datablock(s) I. DOI: 10.1107/S1600536811031771/ci5196Isup2.hkl

Additional supplementary materials:  crystallographic information; 3D view; checkCIF report

## Figures and Tables

**Table 1:** Selected bond lengths (Å)

Ni1—N1	1.976 (7)
Ni1—N2	1.981 (7)
Ni1—O1	2.008 (6)
Ni1—O3	2.014 (6)
Ni1—O2	2.132 (5)
Ni1—O4	2.160 (6)

**Table 2:** Hydrogen-bond geometry (Å, °)

*D*—H⋯*A*	*D*—H	H⋯*A*	*D*⋯*A*	*D*—H⋯*A*
O5—H5*B*⋯O1	0.85 (1)	2.22 (7)	2.898 (8)	136 (8)
O5—H5*A*⋯Br2^i^	0.85 (1)	2.92 (5)	3.666 (9)	146 (8)
O4—H4*A*⋯Br1^ii^	0.93	2.90	3.532 (6)	126
O4—H4*A*⋯O5^i^	0.93	2.16	2.841 (9)	130
O2—H2*A*⋯O3^iii^	0.93	1.97	2.694 (7)	133

Symmetry codes: (i) 
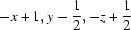
; (ii) 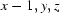; (iii) 
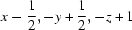
.

**Fig. 1.**
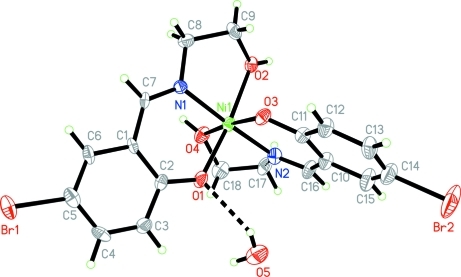
The molecular structure of the title compound, showing the atom-numbering scheme. Displacement ellipsoids are drawn at the 30% probability level. The dashed line indicates a hydrogen bond.

**Fig. 2.**
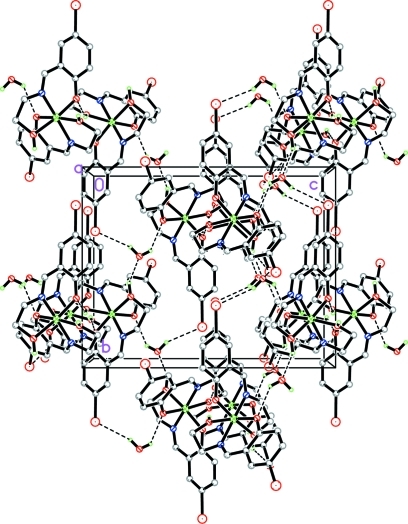
The crystal packing of the title compound. Hydrogen bonds are shown as dashed lines.

## supplementary crystallographic information

### Comment

As part of our investigations into novel urease inhibitors (Wang & Ye, 2011; Wang, 2009), we have synthesized the title compound, a new mononuclear nickel(II) complex, Fig. 1. The compound contains a mononuclear nickel(II) complex molecule and a water molecule of crystallization. The Ni atom in the complex is six-coordinated by two phenolate O, two imine N, and two hydroxy O atoms from two Schiff base ligands, forming an octahedral geometry. The *trans* angles at the Ni atom are in the range 173.1 (3)–174.0 (2)°; the other angles are close to 90°, ranging from 80.4 (3) to 94.8 (2)°, indicating a slightly distorted octahedral coordination. The Ni—O and Ni—N bond lengths (Table 1) are typical and are comparable to those observed in other similar nickel(II) complexes (Arıcı *et al.*, 2005; Liu *et al.*, 2006; Li & Wang, 2007; Ali *et al.*, 2006).

In the crystal structure of the compound, molecules are linked through intermolecular O—H···O and O—H···Br hydrogen bonds (Table 2), to form a three-dimensional network (Fig. 2).

### Experimental

5-Bromosalicylaldehyde (1.0 mmol, 0.201 g), 2-aminoethanol (1.0 mmol, 0.061 g), and nickel nitrate hexahydrate (0.5 mmol, 0.146 g) were dissolved in MeOH (30 ml). The mixture was stirred at room temperature for 10 min to give a clear green solution. After keeping the solution in air for a week, green block-shaped crystals were formed at the bottom of the vessel.

### Refinement

The water H atoms were located in a difference Fourier map and refined isotropically, with O–H and H···H distances restrained to 0.85 (1) and 1.37 (2) Å, respectively. The remaining H atoms were placed in geometrically idealized positions and constrained to ride on their parent atoms, with C–H distances in the range 0.93–0.97 Å, O—H distance of 0.93 Å, and with *U*_iso_(H) set at 1.2*U*_eq_(C and O).

### Crystal data

**Table:**

[Ni(C_9_H_9_BrNO_2_)_2_]·H_2_O	*F*(000) = 1120
*M**_r_* = 562.89	*D*_x_ = 1.823 Mg m^−^^3^
Orthorhombic, *P*2_1_2_1_2_1_	Mo *K*α radiation, λ = 0.71073 Å
Hall symbol: P 2ac 2ab	Cell parameters from 2063 reflections
*a* = 9.835 (3) Å	θ = 2.5–25.3°
*b* = 12.851 (2) Å	µ = 4.87 mm^−^^1^
*c* = 16.226 (3) Å	*T* = 298 K
*V* = 2050.8 (8) Å^3^	BlocK, green
*Z* = 4	0.21 × 0.20 × 0.20 mm

### Data collection

**Table:**

Bruker SMART CCD area-detector diffractometer	4474 independent reflections
Radiation source: fine-focus sealed tube	2310 reflections with *I* > 2σ(*I*)
graphite	*R*_int_ = 0.099
ω scans	θ_max_ = 27.0°, θ_min_ = 2.4°
Absorption correction: multi-scan (*SADABS*; Sheldrick, 1996)	*h* = −12→12
*T*_min_ = 0.428, *T*_max_ = 0.442	*k* = −16→16
13318 measured reflections	*l* = −20→15

### Refinement

**Table:**

Refinement on *F*^2^	Secondary atom site location: difference Fourier map
Least-squares matrix: full	Hydrogen site location: inferred from neighbouring sites
*R*[*F*^2^ > 2σ(*F*^2^)] = 0.048	H atoms treated by a mixture of independent and constrained refinement
*wR*(*F*^2^) = 0.128	*w* = 1/[σ^2^(*F*_o_^2^) + (0.0374*P*)^2^ + 1.9512*P*] where *P* = (*F*_o_^2^ + 2*F*_c_^2^)/3
*S* = 1.02	(Δ/σ)_max_ = 0.001
4474 reflections	Δρ_max_ = 0.60 e Å^−^^3^
259 parameters	Δρ_min_ = −0.95 e Å^−^^3^
3 restraints	Absolute structure: Flack (1983), 1930 Friedel pairs
Primary atom site location: structure-invariant direct methods	Flack parameter: 0.013 (19)

### Special details

**Table:**

Geometry. All e.s.d.'s (except the e.s.d. in the dihedral angle between two l.s. planes) are estimated using the full covariance matrix. The cell e.s.d.'s are taken into account individually in the estimation of e.s.d.'s in distances, angles and torsion angles; correlations between e.s.d.'s in cell parameters are only used when they are defined by crystal symmetry. An approximate (isotropic) treatment of cell e.s.d.'s is used for estimating e.s.d.'s involving l.s. planes.
Refinement. Refinement of *F*^2^ against ALL reflections. The weighted *R*-factor *wR* and goodness of fit *S* are based on *F*^2^, conventional *R*-factors *R* are based on *F*, with *F* set to zero for negative *F*^2^. The threshold expression of *F*^2^ > σ(*F*^2^) is used only for calculating *R*-factors(gt) *etc*. and is not relevant to the choice of reflections for refinement. *R*-factors based on *F*^2^ are statistically about twice as large as those based on *F*, and *R*- factors based on ALL data will be even larger.

### Fractional atomic coordinates and isotropic or equivalent isotropic displacement parameters (Å^2^)

**Table:**

	*x*	*y*	*z*	*U*_iso_*/*U*_eq_	
Br1	1.08191 (9)	−0.02277 (7)	0.25079 (7)	0.0550 (3)	
Br2	0.5363 (2)	0.81053 (8)	0.47258 (10)	0.1150 (7)	
Ni1	0.45998 (10)	0.24321 (8)	0.40117 (7)	0.0323 (3)	
O1	0.6019 (5)	0.2555 (4)	0.3127 (4)	0.0425 (15)	
O2	0.3198 (5)	0.2157 (4)	0.4990 (3)	0.0364 (16)	
H2A	0.2280	0.2329	0.4989	0.044*	
O3	0.5614 (6)	0.3443 (4)	0.4733 (4)	0.0366 (15)	
O4	0.3367 (6)	0.1508 (4)	0.3188 (4)	0.0451 (17)	
H4A	0.3131	0.0812	0.3251	0.054*	
O5	0.5810 (10)	0.4431 (5)	0.2144 (5)	0.074 (2)	
N1	0.5494 (7)	0.1225 (5)	0.4536 (4)	0.0307 (17)	
N2	0.3496 (7)	0.3550 (5)	0.3502 (5)	0.0371 (19)	
C1	0.7389 (8)	0.1086 (6)	0.3572 (5)	0.029 (2)	
C2	0.7047 (8)	0.1925 (6)	0.3039 (5)	0.032 (2)	
C3	0.7945 (8)	0.2085 (6)	0.2369 (6)	0.040 (2)	
H3	0.7772	0.2640	0.2016	0.048*	
C4	0.9041 (9)	0.1480 (7)	0.2209 (6)	0.042 (2)	
H4	0.9582	0.1617	0.1752	0.051*	
C5	0.9358 (9)	0.0658 (7)	0.2726 (6)	0.042 (2)	
C6	0.8539 (8)	0.0483 (6)	0.3384 (6)	0.038 (2)	
H6	0.8751	−0.0070	0.3732	0.046*	
C7	0.6629 (8)	0.0814 (6)	0.4292 (5)	0.028 (2)	
H7	0.6986	0.0286	0.4620	0.033*	
C8	0.4827 (8)	0.0827 (6)	0.5276 (5)	0.040 (2)	
H8A	0.5507	0.0620	0.5676	0.047*	
H8B	0.4287	0.0220	0.5138	0.047*	
C9	0.3914 (9)	0.1664 (7)	0.5646 (6)	0.045 (3)	
H9A	0.3276	0.1353	0.6030	0.054*	
H9B	0.4458	0.2171	0.5941	0.054*	
C10	0.4558 (9)	0.5003 (6)	0.4176 (5)	0.041 (2)	
C11	0.5478 (8)	0.4458 (6)	0.4704 (5)	0.033 (2)	
C12	0.6275 (8)	0.5055 (6)	0.5233 (6)	0.045 (2)	
H12	0.6845	0.4714	0.5602	0.055*	
C13	0.6258 (10)	0.6126 (7)	0.5234 (6)	0.051 (3)	
H13	0.6819	0.6502	0.5586	0.061*	
C14	0.5408 (13)	0.6614 (7)	0.4712 (7)	0.060 (3)	
C15	0.4569 (12)	0.6097 (7)	0.4200 (7)	0.062 (3)	
H15	0.3987	0.6469	0.3857	0.075*	
C16	0.3650 (8)	0.4525 (6)	0.3608 (6)	0.041 (2)	
H16	0.3121	0.4964	0.3285	0.049*	
C17	0.2488 (9)	0.3159 (7)	0.2904 (6)	0.051 (3)	
H17A	0.2340	0.3675	0.2477	0.061*	
H17B	0.1628	0.3033	0.3180	0.061*	
C18	0.2992 (9)	0.2178 (6)	0.2530 (7)	0.051 (3)	
H18A	0.2286	0.1858	0.2198	0.062*	
H18B	0.3771	0.2316	0.2180	0.062*	
H5A	0.584 (12)	0.395 (5)	0.178 (4)	0.080*	
H5B	0.605 (11)	0.415 (6)	0.260 (3)	0.080*	

### Atomic displacement parameters (Å^2^)

**Table:**

	*U*^11^	*U*^22^	*U*^33^	*U*^12^	*U*^13^	*U*^23^
Br1	0.0343 (4)	0.0641 (6)	0.0666 (7)	0.0118 (5)	0.0052 (6)	−0.0190 (6)
Br2	0.2030 (18)	0.0286 (5)	0.1133 (12)	0.0049 (9)	−0.0640 (13)	−0.0095 (7)
Ni1	0.0245 (5)	0.0278 (5)	0.0446 (7)	0.0024 (5)	0.0003 (5)	0.0031 (5)
O1	0.032 (3)	0.036 (3)	0.059 (4)	0.013 (3)	0.003 (3)	0.011 (3)
O2	0.026 (3)	0.038 (4)	0.045 (4)	0.015 (3)	−0.003 (3)	0.004 (3)
O3	0.030 (4)	0.032 (3)	0.048 (4)	0.001 (3)	−0.006 (3)	0.009 (3)
O4	0.051 (4)	0.035 (3)	0.050 (5)	−0.003 (3)	−0.012 (4)	0.001 (3)
O5	0.076 (5)	0.062 (4)	0.083 (6)	−0.010 (5)	0.002 (5)	0.017 (4)
N1	0.031 (4)	0.025 (3)	0.036 (5)	−0.005 (3)	0.008 (4)	0.000 (3)
N2	0.027 (4)	0.034 (4)	0.050 (5)	−0.005 (3)	−0.002 (4)	−0.005 (4)
C1	0.026 (5)	0.031 (5)	0.029 (6)	0.008 (4)	0.001 (4)	−0.012 (4)
C2	0.030 (5)	0.024 (4)	0.041 (6)	0.000 (4)	−0.006 (5)	−0.002 (4)
C3	0.043 (5)	0.034 (5)	0.044 (7)	−0.002 (4)	0.000 (5)	0.004 (5)
C4	0.029 (5)	0.060 (6)	0.037 (6)	−0.011 (5)	0.013 (4)	−0.006 (5)
C5	0.034 (5)	0.045 (5)	0.046 (7)	−0.007 (4)	0.001 (5)	−0.022 (5)
C6	0.027 (5)	0.033 (5)	0.055 (7)	0.006 (4)	0.007 (5)	−0.012 (5)
C7	0.026 (5)	0.019 (4)	0.038 (6)	0.007 (3)	−0.005 (4)	−0.006 (4)
C8	0.036 (5)	0.043 (5)	0.039 (6)	0.001 (4)	0.002 (5)	0.012 (5)
C9	0.031 (5)	0.058 (6)	0.045 (7)	0.013 (4)	0.007 (5)	−0.008 (5)
C10	0.043 (5)	0.034 (4)	0.048 (6)	0.004 (4)	−0.008 (5)	0.000 (4)
C11	0.027 (5)	0.035 (5)	0.036 (6)	−0.001 (4)	−0.008 (5)	0.001 (4)
C12	0.046 (6)	0.038 (5)	0.052 (7)	0.007 (4)	−0.009 (5)	−0.001 (5)
C13	0.057 (7)	0.037 (5)	0.059 (8)	−0.001 (5)	−0.009 (6)	−0.016 (5)
C14	0.080 (8)	0.031 (5)	0.067 (8)	−0.001 (6)	−0.008 (8)	0.001 (5)
C15	0.077 (7)	0.037 (5)	0.073 (8)	0.013 (6)	−0.018 (7)	−0.004 (5)
C16	0.036 (5)	0.029 (5)	0.057 (7)	0.004 (4)	−0.018 (5)	0.002 (5)
C17	0.040 (6)	0.040 (5)	0.072 (8)	0.012 (5)	−0.008 (5)	0.011 (5)
C18	0.048 (5)	0.053 (6)	0.053 (7)	−0.002 (4)	−0.020 (6)	0.000 (6)

### Geometric parameters (Å, °)

**Table:**

Ni1—N1	1.976 (7)	C4—C5	1.385 (12)
Ni1—N2	1.981 (7)	C4—H4	0.93
Ni1—O1	2.008 (6)	C5—C6	1.356 (11)
Ni1—O3	2.014 (6)	C6—H6	0.93
Ni1—O2	2.132 (5)	C7—H7	0.93
Ni1—O4	2.160 (6)	C8—C9	1.523 (11)
Br1—C5	1.867 (9)	C8—H8A	0.97
Br2—C14	1.917 (8)	C8—H8B	0.97
O1—C2	1.303 (9)	C9—H9A	0.97
O2—C9	1.424 (10)	C9—H9B	0.97
O2—H2A	0.9298	C10—C15	1.407 (12)
O3—C11	1.312 (8)	C10—C16	1.423 (11)
O4—C18	1.420 (10)	C10—C11	1.430 (11)
O4—H4A	0.9298	C11—C12	1.392 (11)
O5—H5A	0.853 (10)	C12—C13	1.377 (11)
O5—H5B	0.854 (10)	C12—H12	0.93
N1—C7	1.297 (10)	C13—C14	1.346 (13)
N1—C8	1.461 (10)	C13—H13	0.93
N2—C16	1.274 (10)	C14—C15	1.346 (14)
N2—C17	1.476 (11)	C15—H15	0.93
C1—C6	1.405 (10)	C16—H16	0.93
C1—C2	1.422 (11)	C17—C18	1.485 (12)
C1—C7	1.431 (11)	C17—H17A	0.97
C2—C3	1.416 (12)	C17—H17B	0.97
C3—C4	1.354 (11)	C18—H18A	0.97
C3—H3	0.93	C18—H18B	0.97
			
N1—Ni1—N2	173.1 (3)	N1—C7—C1	126.8 (8)
N1—Ni1—O1	93.4 (2)	N1—C7—H7	116.6
N2—Ni1—O1	91.5 (3)	C1—C7—H7	116.6
N1—Ni1—O3	92.1 (2)	N1—C8—C9	110.0 (7)
N2—Ni1—O3	92.6 (2)	N1—C8—H8A	109.7
O1—Ni1—O3	91.2 (2)	C9—C8—H8A	109.7
N1—Ni1—O2	80.6 (2)	N1—C8—H8B	109.7
N2—Ni1—O2	94.4 (3)	C9—C8—H8B	109.7
O1—Ni1—O2	174.0 (2)	H8A—C8—H8B	108.2
O3—Ni1—O2	89.7 (2)	O2—C9—C8	108.1 (7)
N1—Ni1—O4	94.8 (2)	O2—C9—H9A	110.1
N2—Ni1—O4	80.4 (3)	C8—C9—H9A	110.1
O1—Ni1—O4	89.5 (2)	O2—C9—H9B	110.1
O3—Ni1—O4	173.0 (2)	C8—C9—H9B	110.1
O2—Ni1—O4	90.4 (2)	H9A—C9—H9B	108.4
C2—O1—Ni1	124.7 (5)	C15—C10—C16	117.0 (8)
C9—O2—Ni1	108.1 (5)	C15—C10—C11	117.9 (8)
C9—O2—H2A	126.0	C16—C10—C11	125.0 (7)
Ni1—O2—H2A	125.9	O3—C11—C12	117.9 (7)
C11—O3—Ni1	124.8 (5)	O3—C11—C10	125.0 (7)
C18—O4—Ni1	106.1 (5)	C12—C11—C10	117.1 (7)
C18—O4—H4A	126.9	C13—C12—C11	123.0 (8)
Ni1—O4—H4A	126.9	C13—C12—H12	118.5
H5A—O5—H5B	106 (3)	C11—C12—H12	118.5
C7—N1—C8	119.7 (7)	C14—C13—C12	118.2 (9)
C7—N1—Ni1	124.9 (6)	C14—C13—H13	120.9
C8—N1—Ni1	115.4 (5)	C12—C13—H13	120.9
C16—N2—C17	120.2 (8)	C13—C14—C15	122.6 (9)
C16—N2—Ni1	126.3 (6)	C13—C14—Br2	118.3 (8)
C17—N2—Ni1	113.3 (5)	C15—C14—Br2	119.1 (8)
C6—C1—C2	118.5 (8)	C14—C15—C10	121.0 (9)
C6—C1—C7	117.5 (8)	C14—C15—H15	119.5
C2—C1—C7	123.9 (7)	C10—C15—H15	119.5
O1—C2—C3	118.5 (7)	N2—C16—C10	125.9 (8)
O1—C2—C1	126.0 (8)	N2—C16—H16	117.0
C3—C2—C1	115.4 (7)	C10—C16—H16	117.0
C4—C3—C2	124.0 (8)	N2—C17—C18	109.5 (7)
C4—C3—H3	118.0	N2—C17—H17A	109.8
C2—C3—H3	118.0	C18—C17—H17A	109.8
C3—C4—C5	120.1 (9)	N2—C17—H17B	109.8
C3—C4—H4	119.9	C18—C17—H17B	109.8
C5—C4—H4	119.9	H17A—C17—H17B	108.2
C6—C5—C4	118.0 (8)	O4—C18—C17	107.1 (8)
C6—C5—Br1	120.3 (7)	O4—C18—H18A	110.3
C4—C5—Br1	121.6 (7)	C17—C18—H18A	110.3
C5—C6—C1	123.8 (9)	O4—C18—H18B	110.3
C5—C6—H6	118.1	C17—C18—H18B	110.3
C1—C6—H6	118.1	H18A—C18—H18B	108.6

### Hydrogen-bond geometry (Å, °)

**Table:**

*D*—H···*A*	*D*—H	H···*A*	*D*···*A*	*D*—H···*A*
O5—H5B···O1	0.85 (1)	2.22 (7)	2.898 (8)	136 (8)
O5—H5A···Br2^i^	0.85 (1)	2.92 (5)	3.666 (9)	146 (8)
O4—H4A···Br1^ii^	0.93	2.90	3.532 (6)	126
O4—H4A···O5^i^	0.93	2.16	2.841 (9)	130
O2—H2A···O3^iii^	0.93	1.97	2.694 (7)	133

Symmetry codes: (i) −*x*+1, *y*−1/2, −*z*+1/2; (ii) *x*−1, *y*, *z*; (iii) *x*−1/2, −*y*+1/2, −*z*+1.
